# A Hospital-Based Case-Control Study on Raised Homocysteine Level in Vitiligo Patients and Its Association With Disease Severity

**DOI:** 10.7759/cureus.34772

**Published:** 2023-02-08

**Authors:** Nidhi Choudhary, Narendra S Patel, Ajay S Raghuwanshi, Nishant Choudhary, Surbhi Agrawal

**Affiliations:** 1 Dermatology, RKDF Medical College Hospital and Research Centre, Bhopal, IND; 2 Forensic Medicine, Atal Bihari Vajpayee Government Medical College, Vidisha, IND; 3 Dermatology, LN Medical College and Research Centre, Bhopal, IND

**Keywords:** depigmentation, case-control, pigmentary disorder, homocysteine, vitiligo

## Abstract

Introduction

Vitiligo is an acquired pigmentary disorder clinically manifested by circumscribed depigmented macules and often associated with leucotrichia. Not much is known about the biochemical abnormality occurring in vitiligo. Our study aims to determine whether serum homocysteine is raised in vitiligo patients and whether it can be used as a prognostic marker for vitiligo.

Material and methods

This study is a hospital-based, case-control, analytical study conducted on 70 patients of vitiligo patients. A total of 30 staff of the hospital served as control. Venous blood was withdrawn from the antecubital vein from all study participants using all aseptic precautions. Investigation of blood homocysteine levels was done in all the study participants. Scoring of vitiligo was done based on Vitiligo European Task Force (VETF) criteria which take into account body surface area, stage, and spread.

Results

Mean serum homocysteine level among vitiligo patients was 14.40± 5.80 micromoles/lit as compared to 10.33± 5.05 micromole/lit in control groups, and this difference was statistically significant (t-value = 3.19and p-value = 0.002). The correlation coefficient was statistically significant (correlation coefficient = 0.25 and p-value = 0.03) in between homocysteine level and stage of the disease. On multiple comparisons difference in serum homocysteine level of progressing category is significantly raised as compared to control, stable, and regressing categories.

Conclusion

The mean serum homocysteine level among all vitiligo patients was higher as compared to control groups. Moreover, the serum homocysteine level of active cases is significantly higher as compared to control, stable, and regressing categories. Also, serum homocysteine levels showed a positive correlation with the degree of depigmentation, i.e., stage of the disease.

## Introduction

Vitiligo is an acquired pigmentary disorder clinically manifested by circumscribed depigmented macules and often associated with leucotrichia, i.e., whitening of hair. About 0.5%-2% of the world's population is affected by vitiligo [1]. The highest incidence of vitiligo has been recorded in India and Mexico. Though males and females are equally affected, a female preponderance is often noticed, probably because more females seek medical help for this problem. Onset is usually in the first or second decade.

Vitiligo is a multifactorial disorder related to both genetic and non-genetic factors. Various theories have been proposed regarding the pathogenesis of vitiligo. The classic theories include the autoimmune destruction of melanocytes, the destruction of melanocytes by neurochemical substances, and self-destruct theory of Lerner [2].

Autoimmune destruction of melanocytes is the most recognized hypothesis and is supported by the fact that vitiligo patients often have other autoimmune diseases like hypothyroidism, diabetes mellitus, alopecia areata, pernicious anemia, etc.

Various cytokine changes, like a rise in IL-6 and TNF-alpha and a reduction in IFN-gamma, have been found, which are suggestive of the autoimmune nature of the disease [3].

Not much was known about the biochemical abnormality occurring in vitiligo. Recently various workers have demonstrated raised homocysteine, reduced folic acid, and vitamin B12 in the serum of vitiligo patients [4,5].

Vitamin B12 and folic acid are essential cofactors required in homocysteine metabolism. A deficiency of vitamin B12 and folic acid causes homocysteine levels to increase in circulation. Elevated homocysteine level causes oxidative stress on melanocytes by producing reactive oxygen species [6]. Further, there are reports that homocysteine inhibits tyrosinase activity by binding with copper in its active site, resulting in reversible hypopigmentation [7]. Improvement of this disease after treatment with vitamin B12 and folic acid has also been reported [8]. Our study aims to determine whether serum homocysteine is raised in vitiligo patients and whether it can be used as a prognostic marker for vitiligo.

## Materials and methods

This study is a hospital-based, case-control, analytical study conducted at the dermatology outpatient department (OPD) at a tertiary care center in eastern India. The study was of one-year duration. A total of 70 patients of vitiligo of both sexes above the age of 18 years coming to the OPD were recruited. The diagnosis of vitiligo was made solely based on the clinical examination of the patient. Informed consent was taken from every patient. Proper history and analysis of various clinical aspects of the disease were made in great detail in daylight.

Inclusion criteria for patients included patients who were 18 years and above of either sex who had not taken any treatment for the last three months and with no systemic disease known to be associated with raised homocysteine level, including hypertension, cardiovascular disease, renal failure, psoriasis, inflammatory bowel disease, etc. Patients below 18 years of age, with systemic diseases known to be associated with elevated homocysteine levels, or who are unable or unwilling to provide informed consent for any reason were excluded from the study. The patients were screened and recruited to meet up the inclusion criteria. Data of all the patients recruited were filled in a case record form. A total of 30 healthy controls were recruited in the study, who belonged to the age group 25 to 35 years. After obtaining informed written consent from the patients and the controls, 5 ml of venous blood was withdrawn from the antecubital vein using all aseptic precautions. Investigation of blood homocysteine levels in vitiligo patients was done in the biochemistry department by enzyme immune-essay method.

Data were analyzed by appropriate statistical software. Scoring of vitiligo was done based on VETF (Vitiligo European Task Force criteria). VETF is a system that incorporates three components of vitiligo: extent, stage, and progression of the disease [9]. The extent is based on the rule of nines. The staging was done by assessing the largest macule in each area for the degree of depigmentation of skin and hair using a Wood’s lamp when required.

Stage 0: Normal pigmentation (no depigmentation in area graded)

Stage1: Incomplete depigmentation (including spotty depigmentation, trichrome, and homogeneous lighter pigmentation, a few white hair)

Stage 2: Complete depigmentation (a few white hair do not change grading)

Stage 3: Complete depigmentation plus hair whitening <30%

Stage 4: Complete depigmentation with complete hair whitening

Spread was assessed based on the patient’s opinion about the progression or regression of the largest macule of an area in the last year. Spread was categorized as progressive (score +1), stable (score 0), and regressive (score -1). Values were put in a scoring table for different body sites, and the total was calculated separately for each area, staging, and spread.

## Results

A total of 70 vitiligo patients were recruited for the study; out of them, 23 were male, while 47 were female. The average age of presentation was 33.11 years. The majority had the onset of vitiligo in the second decade. Vitiligo vulgaris is the most common type, found in 40% of patients, while vitiligo universalis is the least common type, found in only 1.4% of patients. The maximum serum homocysteine level among vitiligo patients was found to be 29.3 micromole/lit, and the minimum was 3.1 micromole/lit. A total of 30 healthy controls were taken who were all in the age group of 25-35 years. The maximum serum homocysteine level among controls was found to be 22.8 micromole/lit, and the minimum was 2.8 micromole/lit. The mean serum homocysteine level among vitiligo patients was 14.40± 5.80 micromoles/lit as compared to 10.33± 5.05 micromole/lit in control groups, and this difference was statistically significant (t-value = 3.19 and p-value = 0.002).

**Figure 1:**
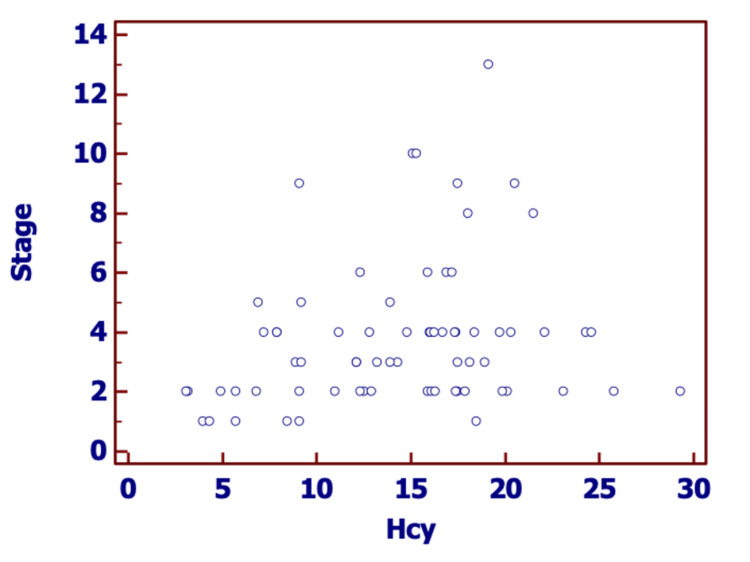
Scatterplot between Homocysteine level in micromole/lit on the x-axis and stage score on the y-axis

Spearman's coefficient of rank correlation between the homocysteine level of patients and their body surface area was 0.137 (95% CI = -0.102 to 0.360), but this relationship was not statistically significant (p-value= 0.25). The correlation coefficient was statistically significant (correlation coefficient = 0.251, 95% CI = 0.0172 to 0.459, p-value = 0.0360) in between the homocysteine level and stage score of the disease (Figure 1).

Spread was categorized as progressive (n=40), stable (n=25), and regressive (n=5), and a fourth-category control group (n=30) was analyzed for differences in their mean Homocysteine levels. Homocysteine parameters among these groups are provided in Table 1.

**Table 1:** Homocysteine level parameters in disease spread categories

Spread Category	Homocysteine level in micromole/lit				
	Mean (SD)	95% Confidence Interval for Mean		Minimum	Maximum
		Lower Bound	Upper Bound		
Control (n=30)	10.5 (5.0)	8.624	12.397	2.8	22.8
Regressing (n=5)	8.1 (4.7)	2.268	14.088	3.2	13.9
Stable (n=25)	12.3 (5.5)	10.072	14.667	3.1	23.1
Progressing (n=40)	16.4 (5.1)	14.815	18.093	4.9	29.3

On multiple comparisons of mean homocysteine levels by Turkey’s test among various disease categories, it is apparent that the difference in serum homocysteine level of progressing category is significantly raised as compared to control, stable, and regressing categories. There was no significant difference found in the serum homocysteine level between the control, stable, and regressing categories (Table 2).

**Table 2:** Multiple comparisons of mean Homocysteine level by Turkey’s test among various disease categories *Significant difference C: Control, R: Regressing, S: Stable, P: Progressing

Spread Category (I)	Spread Category (J)	Mean Difference (I-J)	Std. Error	Sig. (p-value)	95% Confidence Interval	
					Lower Bound	Upper Bound
C	R	2.3323	2.5132	0.790	-4.239	8.903
	S	-1.8593	1.4089	0.553	-5.543	1.825
	P	-5.9437*	1.2566	<0.001*	-9.229	-2.658
R	C	-2.3323	2.5132	0.790	-8.903	4.239
	S	-4.1916	2.5488	0.359	-10.856	2.473
	P	-8.2760*	2.4679	0.006*	-14.729	-1.823
S	C	1.8593	1.4089	0.553	-1.825	5.543
	R	4.1916	2.5488	0.359	-2.473	10.856
	P	-4.0844*	1.3265	0.014*	-7.553	-.616
P	C	5.9437*	1.2566	<0.001*	2.658	9.229
	R	8.2760*	2.4679	0.006*	1.823	14.729
	S	4.0844*	1.3265	0.014*	.616	7.553

## Discussion

As such, there is no skin or serological test to determine the stability or activity of a vitiligo lesion. Grimes reported decreased T helper/suppressor ratio in patients with vitiligo of less than one-year duration [10]. Cui et al. observed cytotoxic antibodies are more common in active vitiligo patients [11].

The production of pro-inflammatory cytokines, like IL-6 and IL-2, and serum homocysteine was seen to be increased in a study by Singh et al. in vitiligo patients [3]. Homocysteine level was reported to be higher in active vitiligo patients than in stable ones, and the mean value of serum folic acid and vitamin B12 was significantly decreased in the vitiligo group as compared to controls [5].

In our study, the mean serum homocysteine level of patients was 14.40±5.80 micromole/lit, while in the control group was 10.33±5.05 micromole/lit. The difference was significant with p < 0.05.

Similarly, Singh et al. also reported a significantly higher level of mean serum homocysteine level in the patient group as compared to the control group (28.8±7.7 vs. 23.1±1.9 p< 0.05) [5]. The difference in serum homocysteine in our research and Singh et al. (14.4±5.80 micromole/lit vs. 28.8±7.7 micromole/lit in patients and 10.33±5.05 micromole/lit. vs. 23.1±1.9 micromole/lit in controls) may be because of variation in ethnicity.

Agarwal et al. too reported a significantly raised mean serum homocysteine level in vitiligo patients than controls (15.39 ± 7.2 mol/L in patients vs. 11.88 ± 4.81 μmol/L in controls P = 0.02) [12].

A significantly higher mean serum level of homocysteine was seen in patients with vitiligo than in controls (21·61 ± 13·28 vs. 13·1 ± 4·88 μmol/ L; P< 0·001) in a study by Shaker et al. [4].

Our result was in contrast to a case-control study which reported no significant difference in the serum homocysteine level between patients and controls [13].

While comparing the serum homocysteine level in regressing, stable, and progressing vitiligo patients, we found that the regressing group had mean values of 8.17±4.7 micromole/lit, the stable group had 12.37±5.5 micromole/lit and progressing group had 16.45±5.12micromol/lit. The difference between the progressive category versus stable and regressive categories was significant, with p<0.05.

Singh et al. also reported a significant increase (p= 0.007) in serum homocysteine levels in active vitiligo patients (30.2±6.5micromol/lit) as compared to stable vitiligo patients (22.8±7.4) [5].

Similarly, a positive correlation between homocysteine level and the activity of vitiligo was reported by Shaker and El-Tahlawi. He also reported significantly higher levels of homocysteine in patients with progressive disease than in controls (25·4 ± 14·99 vs. 13·1 ± 4·88 μmol/ L; P< 0·001) [4].

A significant correlation was found between the staging of vitiligo, i.e., degree of depigmentation and serum homocysteine level with p=0.0360. No studies correlating serum homocysteine levels with the degree of depigmentation were found.

No significant association was found between serum homocysteine level and the percentage of body surface area involvement in vitiligo (p=0.25). These results were in contrast to various studies which reported a positive association of serum homocysteine with the VASI score [14,15].

The limitation of our study is that due to the small sample size, the results cannot be generalized. Due to the limitation of resources, correlation with genetic markers was not done. Further studies with a larger sample size are needed to further prove the point.

## Conclusions

An institution-based, case-control, analytical, controlled study was done to evaluate serum homocysteine levels in 70 vitiligo patients. The serum homocysteine levels were then correlated with the extent, activity, and staging of vitiligo. Thirty healthy controls were selected whose serum homocysteine was compared with the study population. The difference in values of mean serum homocysteine level between the patient and control was significant.

A significant correlation was found between the degree of depigmentation, i.e., the spread of vitiligo, with serum homocysteine level. Also, there was a positive correlation between serum homocysteine and disease activity. The study suggests that there is a role of homocysteine in the etiopathogenesis of vitiligo and that the level of serum homocysteine correlates well with the stage and activity of vitiligo. Hence serum homocysteine level may act as a marker of disease severity and activity of vitiligo. Further studies with a larger sample size are needed to further prove the point.
